# Inhibition of Ghrelin Activity by Receptor Antagonist [d-Lys-3] GHRP-6 Attenuates Alcohol-Induced Hepatic Steatosis by Regulating Hepatic Lipid Metabolism

**DOI:** 10.3390/biom9100517

**Published:** 2019-09-21

**Authors:** Karuna Rasineni, Jacy L. Kubik, Carol A. Casey, Kusum K. Kharbanda

**Affiliations:** 1 Department of Internal Medicine, University of Nebraska Medical Center, Omaha, NE 68198, USA; jlkubik@unmc.edu (J.L.K.); ccasey@unmc.edu (C.A.C.); kkharbanda@unmc.edu (K.K.K.); 2 Research Service, Veterans’ Affairs Nebraska-Western Iowa Health Care System, Omaha, NE 68105, USA

**Keywords:** alcoholic fatty liver, insulin, ghrelin, adipose tissue, [d-Lys-3] GHRP-6

## Abstract

Alcoholic steatosis, characterized by an accumulation of triglycerides in hepatocytes, is one of the earliest pathological changes in the progression of alcoholic liver disease. In our previous study, we showed that alcohol-induced increase in serum ghrelin levels impair insulin secretion from pancreatic β-cells. The consequent reduction in the circulating insulin levels promote adipose-derived fatty acid mobilization to ultimately contribute to hepatic steatosis. In this study, we determined whether inhibition of ghrelin activity in chronic alcohol-fed rats could improve hepatic lipid homeostasis at the pancreas–adipose–liver axis. Adult Wistar rats were fed Lieber-DeCarli control or an ethanol liquid diet for 7 weeks. At 6 weeks, a subset of rats in each group were injected with either saline or ghrelin receptor antagonist, [d-Lys-3] GHRP-6 (DLys; 9 mg/kg body weight) for 5 days and all rats were sacrificed 2 days later. DLys treatment of ethanol rats improved pancreatic insulin secretion, normalized serum insulin levels, and the adipose lipid metabolism, as evidenced by the decreased serum free fatty acids (FFA). DLys treatment of ethanol rats also significantly decreased the circulating FFA uptake, de novo hepatic fatty acid synthesis ultimately attenuating alcoholic steatosis. To summarize, inhibition of ghrelin activity reduced alcoholic steatosis by improving insulin secretion, normalizing serum insulin levels, inhibiting adipose lipolysis, and preventing fatty acid uptake and synthesis in the liver. Our studies provided new insights on the important role of ghrelin in modulating the pancreas–adipose–liver, and promoting adipocyte lipolysis and hepatic steatosis. The findings offer a therapeutic approach of not only preventing alcoholic liver injury but also treating it.

## 1. Introduction

Alcohol abuse is a serious problem in US and worldwide. Among alcoholics, 90% of the people develop alcoholic fatty liver [1], which is characterized by an accumulation of lipids in hepatocytes. The accumulation of fat in hepatocytes makes the liver susceptible to inflammatory mediators or toxic agents, leading to further progression to hepatitis and eventually fibrosis [2,3]. Pathophysiological mechanisms involved in the development of alcoholic fatty liver disease include reduced very-low density lipoprotein (VLDL) secretion, decreased fatty acid oxidation, and increased hepatocyte triglyceride synthesis, mainly as a result of increased uptake and esterification of circulating fatty acids [4,5,6,7]. Recent evidence indicates that alcohol-induced increases in circulating fatty acids is from their enhanced mobilization in adipose tissue [8,9]. Further studies have shown that impaired insulin signaling contributes to increased alcohol-induced triglyceride lipolysis of the adipose tissue [8,10,11]. Impaired insulin-dependent signal transduction has extensive and devastating effects on other organs such as the liver, where it inhibits the export of fats out of the liver, such as VLDL [12,13].

Interestingly, in our recently published study, we observed that chronic consumption of alcohol results in increased ghrelin and decreased serum insulin levels, with a concomitant and significant accumulation of intracellular insulin in pancreatic islets [14]. These observations suggested that the alcohol-induced increase in ghrelin might have impaired the insulin secretion to cause reduced insulin serum levels in the ethanol-fed rats. This conjecture was based on the reported function of ghrelin, a hormone mainly secreted from stomach, to inhibit insulin secretion from pancreatic β-cells, via binding to its receptor GHS-R1a (growth hormone secretagogue receptor type 1a (GHS-R1a) and inhibiting Ca2^+^ influx. In our study we confirmed that alcohol-induced increased serum ghrelin levels indeed decreased the pancreatic insulin secretion, since in vitro addition of ghrelin to ethanol-treated INS-1E pancreatic β-cells and ex-vivo addition of pancreatic islets isolated from control and ethanol-fed rats significantly inhibited the insulin secretion [14]. Since GHS-R1a is distributed in many tissues, including the liver [15,16], we further examined the effects of ghrelin and showed that it can directly promote fat accumulation in hepatocytes [14]. Thus, ghrelin indirectly (by inhibiting insulin secretion, and consequently increasing adipose tissue lipolysis and mobilization of fatty acids to the liver) and directly (by enhancing circulating fatty acid uptake, fatty acid synthesis, and their subsequent esterification) promoted triglyceride accumulation in hepatocytes. Based on these considerations, we postulated that modulating ghrelin synthesis or its activity could be a favorable therapeutic approach for treating alcoholic fatty liver disease.

One of the strategies to inhibit the ghrelin activity is to utilize different ghrelin receptor antagonists [17]. We hypothesized that treatment with a selective ghrelin receptor antagonist will prevent the alcohol-induced effect on pancreas, adipose tissue, and the liver. [D-Lys3]-GHRP-6 (DLys) is one such selective GHS-R1a antagonist that is widely used in in vivo and in vitro [17,18,19,20]. In this study, we examined its effect on alcohol-induced fatty liver disease and further investigated the underlying mechanisms.

## 2. Materials and Methods

### 2.1. Antibodies and Reagents

Antibodies and reagents were purchased from the following companies. Ethanol was purchased from Pharmaco-AAPER (Brookfield, CT, USA). Ghrelin receptor antagonist ([D-Lys3]-GHRP-6) was purchased from Tocris Bio-Techne brand (Minneapolis, MN, USA). Antibodies to AKT, pAKT, HSL, pHSL, AKT, pAKT were purchased from Cell Signaling (Danvers, MA, USA). IRDye infrared secondary antibodies (Abs) and blocking buffer were bought from Li-COR Biosciences (Lincoln, NE, USA). All other chemicals were obtained from Sigma Chemical Co. (St. Louis, MO, USA), unless stated otherwise.

### 2.2. Animal Maintenance, Treatment, and Tissue Collection

As previously described [21], weight matched Male Wistar rats were pair-fed for 6 weeks with Lieber-DeCarli control and ethanol diet. The diet contains 11% carbohydrates, 18% protein, 35% fat and 36% ethanol of total calories with ethanol being replaced isocalorically with maltodextrin in the control diet [22]. After 6 weeks of feeding, half of the rats in the control group and ethanol-fed group received intraperitoneal injection of DLys, a peptide GHS-R1a antagonist at a dose of 9 mg/kg BW for 5 days while the other half received saline injection [18,19,20]. During the treatment period (5 days), all rats were pair-fed to the ethanol rats being administered the antagonist. After 5 days of treatment (6th day), rats were fasted for 6 h before a glucose tolerance test (GTT) was performed as described [23] after which we resumed the respective dietary regimens. Rats were sacrificed the following day (total 7 weeks of feeding). To maintain the “fed” condition at the time of sacrifice, the rats were fed with fresh control and ethanol diet two hours before sacrifice. Rats were sacrificed by anesthesia with isoflurane at which time we collected blood from vena cava, liver, pancreas, stomach and epididymal adipose for further analysis. All animals received humane care as per the American Association for the Accreditation of Laboratory Animal Care guidelines. All protocols were approved by the Institutional Animal Care and Use Committee at the NWIHCS VA Research Service.

### 2.3. Liver Triglycerides and Serum Non-Esterified Free Fatty Acids (NEFA)

As described previously [14], hepatic lipids were extracted and saponified by the Folch extraction method. After saponification, TG were measured spectrophotometrically using the Infinity Triglycerides reagent (Thermo Fisher Scientific (Middletown, VA, USA). A NEFA-HR (2) diagnostic kit from Wako Life Sciences, Inc. (Mountain View, CA, USA).was used to measure serum NEFA.

### 2.4. Serum Enzymes and Hormones Level

Activities of the serum enzymes, alanine aminotransferase (ALT) and aspartate aminotransferase (ALT), were determined by the clinical laboratory at VA NWIHCS. Serum insulin levels were measured by a rat insulin ELISA kit (Mercodia AB, Uppsala, Sweden; Cat #10-1250-01). Acyl ghrelin in the serum was measured using the rat/mouse ghrelin (active) ELISA kit (EMD Millipore Corporation, USA; Cat # EZRGRA-90K).

### 2.5. Gene Expression Analysis

Total RNA was isolated from liver or adipose tissue using the PureLink RNA Mini Kit (Invitrogen, Carlsbad, CA, USA) and reverse transcribed using the High Capacity cDNA Reverse Transcription Kit from Applied Biosystems (Foster City, CA, USA). Quantitative PCR (qPCR) was performed using a TaqMan Gene Expression assay specific for rat DGAT1 (Rn00584870) and TaqMan Fast Universal PCR Master Mix (Applied Biosystems). SYBR Green qPCR was performed using the specific primers listed in Table 1 from Integrated DNA Technologies (Coralville, IA) with iTaq Universal SYBR Green Supermix (Biorad, Hercules, CA, USA). The ∆∆Ct method was used to determination the fold change using actin in liver and ribosomal phosphoprotein (36B4) in adipose tissue for normalization.

### 2.6. Immunohistochemistry

Paraffin-embedded pancreatic tissue sections (5-µm-thick) were deparaffinized in xylene, rehydrated in ethanol and subjected to antigen retrieval by microwaving in 10 mM sodium citrate buffer (pH 6) for 20 min. After cooling to room temperature, the sections were rinsed in PBS (pH 7.4), permeabilized with 2% Triton X-100/PBS, and blocked for 1 h in 1% BSA/PBS. The sections were incubated overnight at 4 °C with antibodies specific for insulin (1/200 dilution) (Abcam, Cambridge, MA, USA) followed by staining with Alexa Fluor-conjugated secondary antibodies. Images were acquired with an LSM 701 Zeiss Confocal Microscope and staining intensity was quantified at the same threshold limit using ImageJ software (National Institutes of Health, Bethesda, MA, USA).

### 2.7. Western Blot

Tissue homogenates were prepared in ice-cold lysis buffer (50 mM Tris-HCl, 150 mM NaCl, 0.1% SDS, 0.5% sodium deoxycholate, 1% Triton X-100, pH 7.4) containing protease inhibitor cocktail (P2714-1BTL, Sigma, St. Louis, MO, USA). Samples were subjected to 12% SDS-PAGE, transferred to nitrocellulose and proteins were detected by incubation with primary antibodies and their respective secondary antibodies. The intensity of protein bands was quantified using the Odyssey Infrared Imager (LI-COR Bioscience, Lincoln, NE, USA) and associated software followed by normalization to β-actin and expressed relative to the control values.

### 2.8. Statistical Analysis

The results are presented as mean ± SEM. Data were analyzed by a one-way ANOVA, followed by Student’s Newman–Keuls post-hoc test. Comparison between two groups was analyzed using the Student’s *t*-test. A *p*-value < 0.05 was considered to be statistically significant.

## 3. Results

### 3.1. Food Intake, Liver and Body Weights, and Serum Hepatic Markers

In this study, control rats were pair-fed a nutritionally balanced isocaloric diet with ethanol-fed rats for 6 weeks, after which half of the rats in both groups received an intraperitoneal injection of ghrelin receptor antagonist, for 5 days. The other half received the saline injection. Studies have been shown that DLys decreases food intake after 4 h of administration [18,24]. Thus, in this study, during the DLys administration period, all rats (control+saline, ethanol+saline, control+antagonist, ethanol+antagonist) were pair-fed to the ethanol rat being administered the antagonist, to maintain nutritional equality among the 4 rats from each experimental group, denoted as Control, Ethanol, Control+DLys, and Ethanol+DLys. As shown in Table 2, we did not observe significant differences in the average food intake in the ethanol-fed rats before DLys, or at the time of DLys administration. Further, we observed similar body weights among all four experimental groups at weekly intervals and at sacrifice. However, liver weight was significantly higher in ethanol- and the DLys-treated ethanol-fed rats, resulting in an increased liver/body weight in these two groups as compared to control rats (Table 2). Serum ALT and AST, which are markers for liver injury, were significantly higher in ethanol-fed rats compared to the controls. However, the DLys treatment moderately decreased the ALT and AST levels in Ethanol+DLys rats (Table 2).

### 3.2. Ghrelin Receptor Antagonist Abrogated the Ethanol-Induced Impaired Glucose Tolerance by Increasing Serum Insulin Levels via Promotion of Insulin Secretion from the Islets

**Figure 1:**
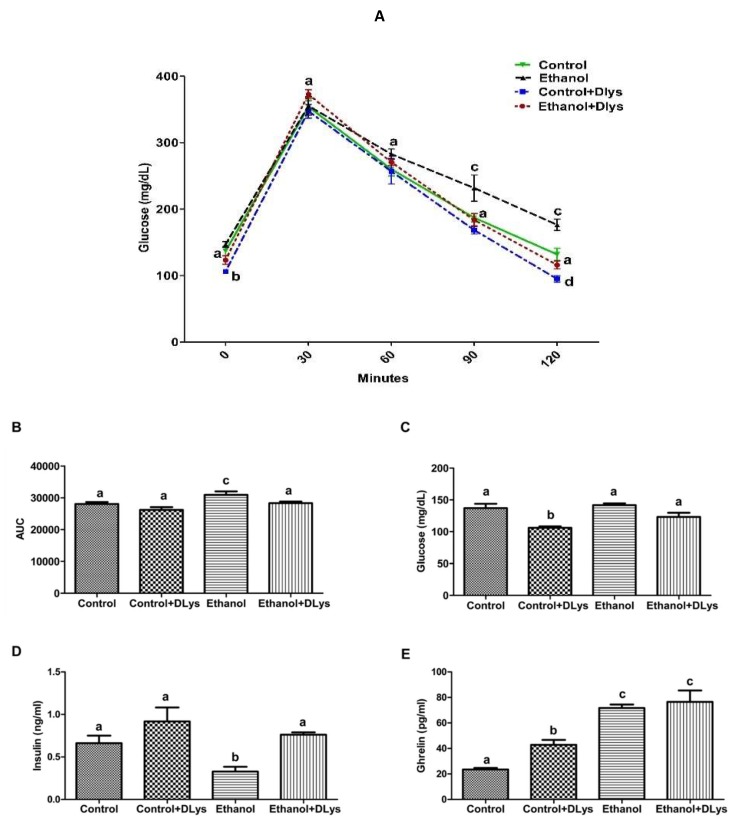
Inhibition of ghrelin activity inhibits the chronic alcohol-induced impaired glucose tolerance and altered serum fasting insulin levels in the rats. (**A**) Glucose tolerance tests (GTT) were performed, as described under experimental procedures. (**B**) Area under the curve (AUC) from GTT. (**C**) Serum glucose. (**D**) Serum insulin. (**E**) Serum ghrelin levels after 6 h fasting. Values are means ± SEM (n = 8–10). Values not sharing a common letter are statistically different, *p* ≤ 0.05.

In our recently published study [14], we had reported that the chronic alcohol administration associated impaired glucose tolerance was because of the alcohol-induced increase in the ghrelin hormone, which impaired insulin secretion from the islets to decrease serum insulin levels. Thus, in this study, we measured fasting insulin levels and conducted GTT after 5 days of DLys treatment. As observed previously, the ethanol-fed rats showed impaired GTT but the Dlys treatment reversed this ethanol-induced effect as the area under curve (AUC) was significantly increased with ethanol consumption (Figure 1A), while similar AUC as control was observed in the DLys-treated ethanol rats (Figure 1B). As observed previously, despite similar blood glucose levels in both groups (Figure 1C), the ethanol-fed rats displayed significant decreases in circulating insulin levels as compared to the control rats (Figure 1D). DLys treatment to the ethanol-fed rats restored the fasting serum insulin levels to the control levels. DLys-treated control rats displayed significant decreases in fasting glucose (Figure 1C) and moderate increases in insulin levels as compared to the saline-treated control rats (Figure 1D). As shown in Figure 1B, the area under curve (AUC) for glucose during GTT for ethanol-fed rats was significantly increased, compared to the control rats, while similar AUC as control was observed in the DLys-treated ethanol rats. These results correlated with the circulating serum insulin levels, which was lower in the ethanol-fed rats as compared to the controls and DLys-treated ethanol rats.

**Figure 2:**
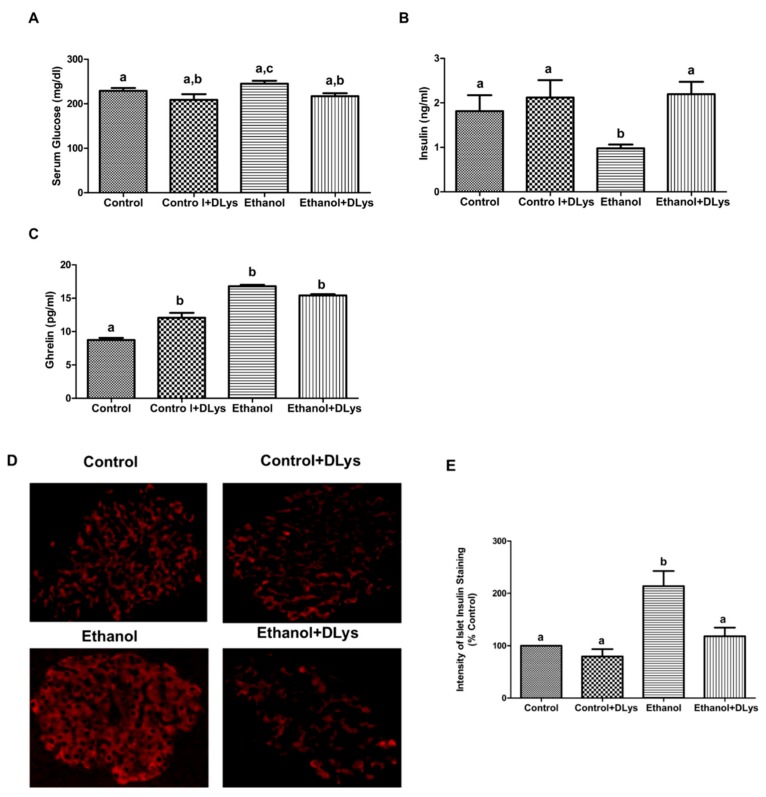
DLys treatment normalizes insulin secretion in ethanol-fed rats. (**A**) Serum glucose; (**B**) serum insulin levels; (**C**) serum ghrelin levels in experimental rats at sacrifice (at the fed condition). (**D**) Immunohistochemistry of insulin in pancreatic islets of experimental rats at the fed-state at a 400X magnification. (**E**) Graphical representation of the intensity/brightness of immunohistochemical staining as a percent of control, demonstrating insulin accumulation in islets of ethanol-fed rats and reduced intensity of insulin staining in islets of DLys-treated, ethanol-fed rats, indicative of normalized insulin secretion by a ghrelin receptor activity inhibition. Values are means ± SEM (n = 8–10). For immunohistochemistry, values are means ± SEM of five individual islets from each section of three sets of control and ethanol-fed animals. Values not sharing a common letter are statistically different, *p* ≤ 0.05.

In addition to measuring the fasting serum insulin, we also measured the serum insulin and ghrelin in the fed condition (blood collected during the sacrifice). We observed similar changes in the serum insulin and ghrelin levels in the saline-treated or the DLys-treated ethanol rats in the “fed conditions” (Figure 2B,C), as seen in the “fasting conditions” (Figure 1D,E). Interestingly, increased serum ghrelin levels were observed in the DLys-treated control rats as compared to the saline-treated control rats, which could be a compensatory effect of the ghrelin receptor inhibition [25]. Note that these increased serum levels correlated with our previously published data, showing that increased serum ghrelin levels are due to an increased ghrelin gene expression in the stomach [14]. However, we observed no significant differences in the protein content of ghrelin receptor (GHS-R) in liver tissues, under any experimental condition (data not shown).

Further, we performed immunostaining for insulin on paraffin sections of the pancreas of rats from all experimental groups. As shown in Figure 2D, we observed a significant accumulation of intracellular insulin in the pancreatic islets of ethanol-fed rats, as we reported before [14]. However, DLys treatment inhibited the ethanol-induced accumulation of insulin in the islets (Figure 2E), which was also reflected by the normalized serum insulin levels (Figure 2B). These results collectively indicated that insulin secretion is restored in ethanol-fed rats to the control levels, when ghrelin action is inhibited.

### 3.3. Inhibition of Ghrelin Activity Attenuates Alcohol-Induced Hepatic Steatosis

**Figure 3:**
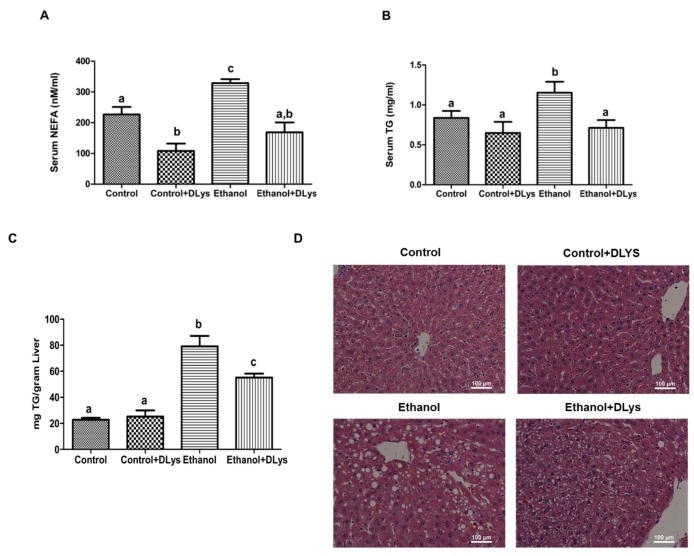
Serum and Hepatic Lipid Content. (**A**) Serum non-esterified free fatty acids (NEFA) levels. (**B**) Serum Triglyceride (TG) levels. (**C**) Quantitative analysis of hepatic TG content. (**D**) Hematoxylin and eosin staining of paraffin sections. Images are representative of each pair-fed group of n = 8. Magnification, 200X. Values are means ± SEM, n = 8. Values not sharing a common letter are statistically different, *p* ≤ 0.05.

At sacrifice, we observed increased serum non-esterified free fatty acids (NEFA) and triglycerides (TG) in ethanol-fed rats, while a receptor antagonist abrogated this ethanol-induced increase (Figure 3A,B). Specifically, as shown in Figure 3A, serum NEFA levels were reduced in both the DLys-treated control and the ethanol-fed rats, as compared to their respective saline-treated groups. Quantitative analysis of hepatic fat showed that chronic alcohol feeding increased the hepatic triglycerides content 3–4 folds (Figure 3C). Histopathological examination of the liver also showed that ethanol administration increased fat accumulation in the liver (Figure 3D). As evident from both quantitative biochemical analysis and histopathological observations, the DLys treatment reduced hepatic triglyceride accumulation in the ethanol-fed rats.

### 3.4. DLys Treatment Abrogates Ethanol-Induced Fat Accumulation via Increasing Hepatic Lipase Activity, Enhancing Fatty acid Oxidation, and Reducing Fatty Acid Synthesis and Uptake

**Figure 4:**
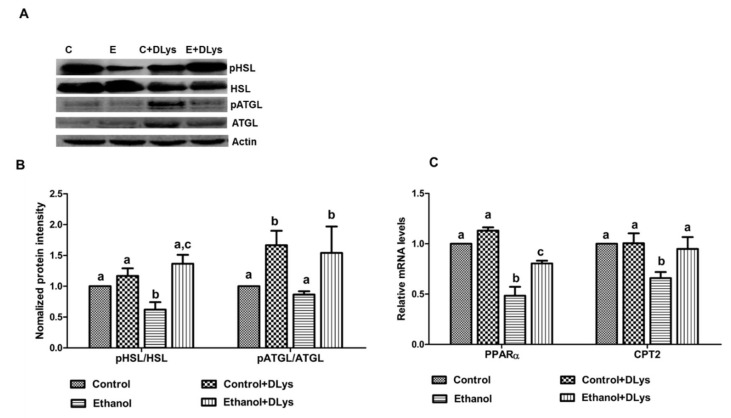
Quantitative analysis of hepatic lipid breakdown and oxidation. (**A**) Representative western blots from control (C), ethanol (E), control+DLys (C+DLys), and ethanol+DLys (E+DLys). (**B**) Densitometric ratios of active (phosphorylated) to total hormone-sensitive lipase (HSL) and adipose triglyceride lipase (ATGL) in the liver of experimental rats. (**C**) qRT–PCR analysis of hepatic gene expression related to fatty acid oxidation-peroxisome proliferator-activated receptor α (PPARα) and carnitine palmitoyltransferase 2 (CPT2). Values are expressed as relative to the control. Values are means ± SEM, n = 8. Values not sharing a common letter are statistically different, *p* ≤ 0.05.

To understand the possible mechanism by which a ghrelin antagonist reduces the alcohol induced fat accumulation in the liver, two main lipases, a hormone sensitive lipase (HSL) and an adipose triacylglycerol lipase (ATGL), which are responsible for a breakdown of triglycerides were measured. We observed a decreased content of the active form of HSL (pHSL in the liver of ethanol-fed rats (Figure 4A,B). In contrast, the DLys treatment caused a significant increase in liver phosphorylated (active) HSL and ATGL, compared to the ethanol-fed rats (Figure 4A,B), indicating that these two active lipases might be contributing to the increased liver triglyceride breakdown in the DLys-treated ethanol rats.

In addition, as shown in Figure 4C, DLys treatment of ethanol-fed rats increased the gene expression of peroxisome proliferator-activated receptor alpha (PPARα), a transcriptional regulator of genes involved in β-oxidation [26] and carnitine palmitoyl transferase II (CPT2), a rate-limiting enzyme of the mitochondrial fatty acid β-oxidation [27]. Increased expression of these genes is likely associated with improved hepatic TG levels in the DLys treated ethanol-fed rats.

**Figure 5:**
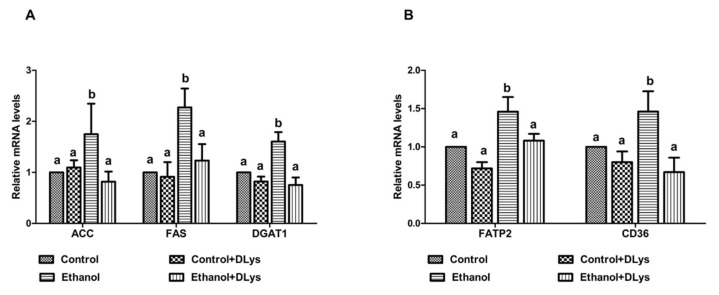
Quantitative analysis of hepatic fatty acid uptake and synthesis. (**A**) qRT–PCR analysis of hepatic gene expression related to fatty acid synthesis-acetyl-CoA carboxylase (ACC), fatty acid synthase (FAS), and diacylglyceride acyltransferase (DGAT1). (**B**) Hepatic gene expression related to fatty acid transport—FATP2 (fatty acid transport protein 2) and fatty acid translocase CD36. Values expressed as a relative to the control values. Values are means ± SEM, n = 8. Values not sharing a common letter are statistically different, *p* ≤ 0.05.

Further, we measured the gene expression of enzymes that are involved in the hepatic fatty acid uptake, synthesis, and esterification. Chronic alcohol administration increased the mRNA levels of enzymes associated with fatty acid synthesis, (fatty acid synthase (FAS), and acetyl CoA carboxylase (ACC)) and esterification (diacylglycerol O-transferase 1 (DGAT1)) (Figure 5A), substantiating the observed increased fat accumulation in ethanol-fed rats (Figure 3C). However, treatment of the ethanol-fed rats with the receptor antagonist, significantly reduced the ethanol-induced hepatic FAS and normalized the ACC and DGAT1 mRNA expression (Figure 5A).

In addition to measuring fatty acid synthesis and esterification, we measured the gene expression of two fatty acid uptake/transport/binding enzymes. Fatty acid transport protein (FATP)2, a member of the FATP family, is a plasma membrane associated long-chain fatty acid transporter. CD36 is a fatty acid translocase, which is also involved in fatty acid uptake. We observed an enhanced expression of FATP2 and CD36 in the liver of ethanol-fed rats as compared to the control rats (Figure 5B). The expression of FATP2 and CD36 was normalized to the control levels in the liver of DLys-treated ethanol-fed rats (Figure 5B).

### 3.5. DLys Treatment Inhibits the Ethanol-Induced Altered Fat Metabolism in the Adipose Tissue

**Figure 6:**
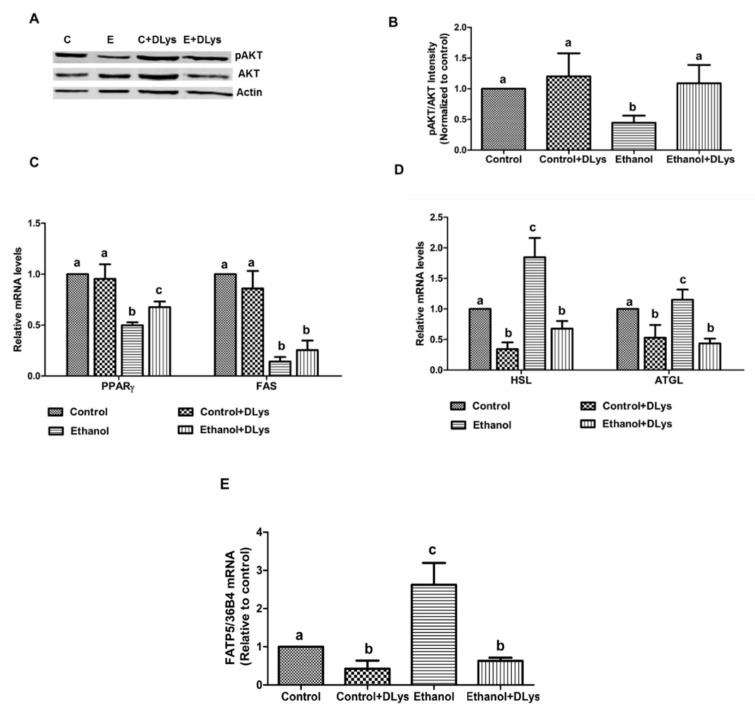
Quantitative analysis of the adipose fatty acid breakdown and transport. (**A**) Representative western blots from the control (C), ethanol (E), control+DLys (C+DLys), and the ethanol+DLys (E+DLys) for total and active AKT. (**B**) Densitometric ratios of active (phosphorylated) to total AKT in the liver of experimental rats. (**C**) qRT–PCR analysis of gene expression related to adipose fatty acid metabolism—peroxisome proliferator-activated receptor γ (PPARγ, a key transcription factor for adipocytes differentiation and expansion) and FAS (fatty acid synthase). (**D**) qRT–PCR analysis of gene expression related to fatty acid breakdown—hormone-sensitive lipase (HSL), adipose triglyceride lipase (ATGL). (**E**) Fatty acid transporter protein 5 (FATP5) to ribosomal protein expression ratio in the adipose tissue of experimental rats. Values are expressed as a relative to the control values. Values are means ± SEM, n = 8. Values not sharing a common letter are statistically different, *p* ≤ 0.05.

Impaired insulin signaling promotes adipose triglyceride breakdown to increase circulating FFA and the development of alcoholic steatosis. Since ghrelin receptor antagonist administration increase the circulating insulin levels, we predicted that the DLys treatment could improve adipose tissue lipid metabolism. To verify this assumption, we measured the lipid metabolism in the epididymal adipose tissue insulin signaling pathway as well as the enzymes related to fatty acid metabolism and transport. Western blot analysis data indicated that ethanol feeding decreased the level of activation (phosphorylation) of AKT/protein kinase B, a downstream target of an insulin signaling cascade involved in the regulation of glucose and lipid metabolism (Figure 6A,B). DLys treatment improved the activation of AKT in the adipose tissue of ethanol-fed rats. Further, we observed that ethanol feeding decreased the expression of peroxisome proliferator-activated receptor-γ (PPARγ), a key player in adipocyte differentiation that was regulated by insulin [28]. As expected with the increased insulin levels, DLys treatment significantly increased PPARγ expression in ethanol-fed rats (Figure 6C). In agreement with the observed ethanol-induced enhanced adipose lipolysis, the mRNA levels of the major adipose lipases, ATGL and HSL were increased significantly in the ethanol-fed rats (Figure 6D). This occurred in conjunction with a significant increase in the expression of fatty acid transporter protein 5 (FATP5) in these rats (Figure 6E), which is indicative of an increased transport of fatty acids from the adipose due to increased lipolysis. DLys treatment to ethanol-fed rats decreased the ATGL and HSL expression in both the control and ethanol-fed rats and also reduced the FATP5 expression, indicating an overall improvement in the adipose lipid metabolism.

## 4. Discussion

Recent investigations from our laboratory and others have demonstrated that chronic alcohol administration reduces serum insulin levels, which in turn leads to enhanced adipose tissue lipolysis to increase the circulating FFA levels. The subsequent delivery and uptake of these fatty acids ultimately results in the development of hepatic steatosis [8,10,11]. More importantly, we showed that the increase in serum ghrelin hormone levels after chronic ethanol consumption is responsible for impaired insulin secretion from the pancreas and the consequences delineated above, which leads to the development of hepatic steatosis [14]. Based on these observations and the central role of ghrelin, we conducted the present study on the premise that ghrelin inhibition could prevent the sequence of events to thereby prevent the development of alcohol-induced liver injury. In this study, we demonstrated that using a ghrelin receptor antagonist restored pancreatic insulin secretion and improved circulating levels that, in turn, inhibited the ethanol-induced increases in the adipose-derived serum FFA and hepatic fat dysregulation to ultimately prevent alcoholic hepatic steatosis.

In this study, as has traditionally been done, the rats in the control group were nutritionally pair-fed with the ethanol-fed rats [29,30]. Since studies been shown that DLys decreases food intake after its administration [18,24], during the DLys treatment, all rats were pair-fed to the ethanol rat being administered the antagonist (Ethanol+DLys group). In addition, we also assessed food intake throughout the study. We observed that ethanol rats exhibited similar calorie intake even after DLys treatments, indicating that inhibition of the ghrelin activity with DLys, at a dose of 9 mg/kg BW did not significantly affect the food intake. Consistent with our previous studies and the recorded food intake in the present study, we observed similar body weights of the ethanol-fed rats, with or without DLys treatment, compared to their pair-fed controls. However, ethanol-fed rats exhibited a significant increase in liver weight and a significant decrease in adipose weight, resulting in an increased liver/body weight ratio and a decreased adipose/body weight ratio as compared to the control rats (Table 2). The DLys treatment did not affect the ethanol-induced changes in adipose and liver weight. Further, while ethanol administration increased the hepatic injury markers, AST and ALT in the serum, DLys treatment partly decreased the ethanol-induced increases in these serum enzymes (Table 2). More importantly, treatment with the antagonist did not induce any further liver damage.

In our previous study, we reported that chronic alcohol administration was associated with a significant decrease in serum insulin and was also associated with an impaired GTT [14]. On the contrary, we observed increased serum levels of the ghrelin hormone. As it is known that ghrelin inhibits insulin secretion [31,32,33], we hypothesized that inhibition of ghrelin activity could improve the serum insulin levels and thereby the GTT. In this current study, as previously reported [14], we observed impaired insulin secretion from the pancreas with a concomitant decrease in serum insulin levels, after chronic alcohol administration. Ghrelin receptor antagonist treatment abrogated the chronic ethanol-induced impaired insulin secretion, as evidenced by similar serum and intracellular islet insulin levels in the DLys-treated ethanol-fed rats as the control rats. Interestingly, antagonist treated control-rats showed increased ghrelin levels as compared with saline-treated control rats. This is likely due to an increased compensatory synthesis of ghrelin, as evidenced by an increased expression of ghrelin in the stomach of the DLys-treated control and ethanol-fed rats (data not shown). This increased expression of ghrelin in the ethanol rats after DLys treatment could be a compensatory mechanism due to the inhibition of ghrelin activity by a receptor antagonist [25].

Consistent with all previous studies, chronic alcohol-administered rats showed nearly three times higher hepatic triglycerides than their pair-fed controls (Figure 3C). This increased accumulation of fats in liver were majorly due to an increased uptake of circulating FFA that were derived from the ethanol-induced enhanced adipose lipolysis. This increase in lipolysis was corroborated by the observed decreases in adipose to the body/weight ratio (Table 2). Clinical studies also demonstrated a negative correlation between liver fat and body fat mass and have shown that alcoholics who have fatty liver have significantly lower body weight and lower fat mass than the controls [34,35]. DLys treatment significantly decreased serum FFA, likely due to regulated serum insulin levels and improved adipose tissue lipid metabolism. In line with these observations, DLys-treated ethanol rats exhibited significantly decreased hepatic triglycerides as compared with the ethanol-fed rats.

Ghrelin has been shown to modulate hepatic lipid metabolism both in vitro and in vivo. Li et al. showed that genetic disruption of either ghrelin or ghrelin receptor genes reduces the incidence of obesity and hepatic steatosis in mice. In addition, they demonstrated that the ghrelin directly increased activation of the mTOR-PPARγ signaling pathway involved in hepatic lipogenesis [16]. Barazzoni et al. [36] also reported that chronic infusion of ghrelin increased hepatic lipid accumulation. In our previous study, we demonstrated that in addition to inhibiting insulin secretion and consequently increasing adipose tissue lipolysis and circulating free fatty acid levels, ghrelin can directly promote hepatic fat accumulation by increasing hepatic uptake of circulating FFA as well as by promoting de novo fatty acid synthesis [14]. We indeed showed that chronic alcohol administration promotes hepatic de novo fatty acid synthesis (as evidenced by FAS and ACC mRNA increase), fatty acid uptake (CD 36 and FATP2 mRNA increase), and esterification (DGAT1 mRNA increase), to ultimately increase the fat accumulation. Inhibition of the ghrelin activity by the DLys treatment normalized the hepatic fatty acid uptake and synthesis to the control levels (Figs. 5A and B), to ultimately prevent the ethanol-induced increased hepatic triglyceride accumulation. The decreased triglyceride accumulation in the livers of ghrelin receptor antagonist-treated ethanol rats could be via a direct inhibition of ghrelin activity in hepatocytes or through a decreased availability of the circulating FFA for hepatic uptake.

It is known that imbalances in the insulin levels and insulin signaling have widespread and devastating effects on many organs, including the liver and the adipose tissue. In the liver, insulin stimulates the export of fatty acids as lipoproteins to the circulation. These lipoproteins are carried into the circulation as VLDL and are taken up by the various organs to provide free fatty acids for use in these tissues, including adipocytes [37]. Adipocytes, in turn, use free fatty acids to synthesize triglycerides that are stored in lipid droplets for energy needs. Insulin inhibits lipolysis in adipocytes, thus, allowing for increased lipid storage in these cells [38,39]. Chronic alcohol-induced increased ghrelin impairs insulin secretion, therefore, stimulates FFA release from adipose tissue, resulting in the promotion of hepatic de novo lipogenesis and development of fatty liver [9,40]. Not surprisingly, increased serum insulin levels in the DLys-treated ethanol-fed rats was associated with decreased serum free fatty acid levels, indicating an improved adipose lipid metabolism in these rats. In addition to serum free fatty acids, we also measured genes involved in lipid synthesis and breakdown in adipose tissue. Alcohol administration decreased PPARγ, which played a crucial role in maintaining adipose expansion and adiposity. DLys treatment increased the adipose PPARγ content (Figure 6C). Several studies demonstrated that PPARγ activation attenuates both alcoholic and non-alcoholic fatty liver injury [41,42]. Further, we observed that alcohol administration decreased FAS mRNA, increased lipid breakdown (increased activation of the two lipases, HSL and ATGL) and increased lipid transport (FATP5 increase) in the adipose tissue. Ghrelin receptor antagonist prevented the abnormal lipid metabolism in adipose tissue by improving lipid synthesis and suppressing lipid breakdown (Figure 6). Since PPARγ promotes adipose differentiation and fat storage in adipocytes, increased PPARγ expression in the DLys-treated ethanol-fed rats could be responsible for increased fat storage. Further improved insulin levels with DLys treatment is mostly responsible for inhibition of adipose lipolysis by inhibiting HSL and ATGL activation.

## 5. Conclusions

**Figure 7:**
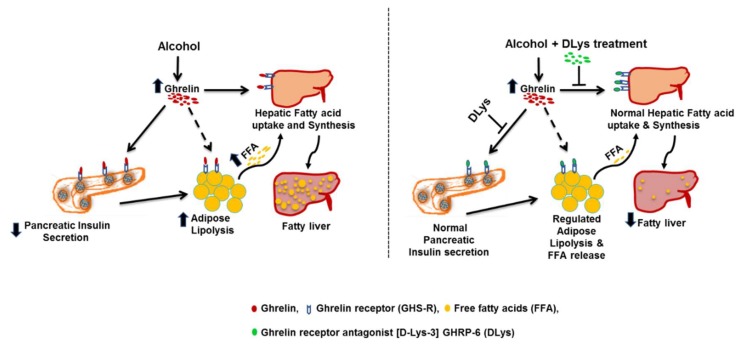
Mechanistic role of ghrelin receptor antagonist in attenuating alcohol-induced hepatic steatosis. Alcohol elevates levels of circulating ghrelin which binds to its receptor, GHS-R, on pancreatic β-cells to impair insulin secretion. The consequent reduction in the circulating insulin levels contribute to increased free fatty acid mobilization from adipose tissue to the liver. Ghrelin also directly act on liver via the ghrelin receptors that are present on hepatocytes, to promote circulating free fatty acid uptake, increase de novo fatty acid synthesis and inhibit fatty acid oxidation, thereby contributing to the development of hepatic steatosis. Inhibition of ghrelin activity by receptor antagonist. [d-Lys-3] GHRP-6 (DLys)-attenuated alcoholic steatosis by improving pancreatic insulin secretion, normalizing serum insulin levels, inhibiting adipose lipolysis, decreasing circulating free fatty acid levels, and preventing de novo fatty acid synthesis in the liver.

To summarize, alcohol-induced elevation of circulating ghrelin levels impairs insulin secretion from the pancreas, thus modulating the adipose–liver axis by promoting adipose tissue lipolysis and increased delivery and uptake of FFA by the liver, to ultimately lead to the development of alcoholic steatosis (Figure 7). Treatment with ghrelin receptor antagonist for only 5 days in the last week of a 7-week chronic ethanol administration, normalized insulin secretion from the pancreas to prevent the downstream consequences and ultimately prevent the development of alcoholic steatosis (Figure 7). Our previous [14] and the current study buttresses the important role of ghrelin in modulating the pancreas–adipose–liver axis and promoting hepatic steatosis, and offers a therapeutic approach of not only preventing alcoholic liver injury but also treating it.

In our future studies, we will also focus on other components that are involved in ghrelin biology, which might interact in the development of alcoholic steatosis, such as (i) the ghrelin O-acyl transferase enzyme that acylates ghrelin, which is required for binding and activating its receptor [43], (ii) the ghrelin antagonist peptide LEAP-2 (liver-expressed antimicrobial peptide-2) which obstructs ghrelin activity by inhibiting ghrelin receptor [44,45], (iii) examining the interaction of GHS-R1 with several other G-protein coupled receptors [46,47], as such co-interactions could lead to either attenuation or augmentation of receptor-mediated signaling. Whether such receptor interactions also exist in the liver and adipose tissues, along with their role in the development of fatty liver disease, will be the focus of our future studies.

## Figures and Tables

**Table 1:** Primer sets used in RT–PCR analysis.

Gene	Forward	Reverse
CPT2	CCCTTAGAGATTCAGGCACATC	TAGGCAGAGGCAGAAGACAGCA
PPARγ	GAGATCCTCCTGTTGACCCAG	CCACAGAGCTGATTCCGAAGT
PPARα	GTCCTCTGGTTGTCCCCTTG	GTCAGTTCACAGGGAAGGCA
FAS	TCCCAGGTCTTGCCGTGC	GCGGATGCCTAGGATGTGTGC
ATGL	CCCGGTTGTCCCCCAGGAAGA	TCCAGCAGGGCCTCGTTGAGT
FATP2	AGT ACA TCG GTG AAC TGC TTC GGT	TGC CTT CAG TGG AAG CGT AGA ACT
FATP5	TTC AGG GAC CAC TGG ACT TCC AAA	ACC ACA TCA TCA GCT GTT CTC CCA
CD36	AACCCAGAGGAAGTGGCAAAG	GACAGTGAAGGCTCAAAGATGG
ACC	TGAGGAGGACCGCATTTATC	GAAGCTTCCTTCGTGACCAG

**Table 2:** Values of the selected parameters at sacrifice.

	Control	Control + DLys	Ethanol	Ethanol + DLys
Food Intake/Day (ml)	Pair-fed	Pair-fed	93 ± 2.93	85 ± 6.32
Body Weight (g)	414 ± 23	423 ± 16	382 ± 18	387 ± 11
Relative Liver Weight (g/100 g Body Weight)	3.03 ± 0.04^a^	3.14 ± 0.09^a^	3.79 ± 0.32^b^	3.54 ± 0.09^b^
Relative Adipose Weight (g/100 g Body Weight)	2.16 ± 0.05^a^	1.93 ± 0.15^a^	1.50 ± 0.13^b^	1.67 ± 0.13^a,b^
Serum ALT (U/L)	52.2 ± 3.97^a^	53.32 ± 1.99^a^	70.76 ± 5.25^b^	60.87 ± 3.75^b^
Serum AST (U/L)	51.2 ± 0.97^a^	56.0 ± 4.82^a^	81.0 ± 6.15^b^	69.8 ± 1.38^b^

Values are means ± SEM (n = 10–14). Values not sharing a common letter are statistically different, *p* ≤ 0.05.
